# Observational study of the effects of age, diabetes mellitus, cirrhosis and chronic kidney disease on sublingual microvascular flow

**DOI:** 10.1186/cc12153

**Published:** 2013-03-19

**Authors:** T Reynolds, S Jhanji, A Vivian-Smith, RM Pearse

**Affiliations:** 1 Royal London Hospital, London, UK; 2 Royal Marsden Hospital, London, UK

## Introduction

Sidestream dark-field (SDF) imaging is an important new technology that has been used to demonstrate microcirculatory abnormalities in a variety of critical illnesses [1]. The microcirculation is also affected by age and chronic comorbidities. However, the effect of these conditions on SDF microcirculatory parameters has not been well described.

## Methods

Sublingual SDF images were obtained from five groups of 20 participants: healthy volunteers under 25 years, healthy volunteers over 55 years, and stable patients over 55 years with one of diabetes mellitus (DM), cirrhosis and stage 5 chronic kidney disease (CKD). Microcirculatory parameters [1] between the groups were then compared for significance using ANOVA for parametric data and the Kruskal-Wallis test for nonparametric data. This was approved by the local ethics committee.

## Results

**Figure 1:**
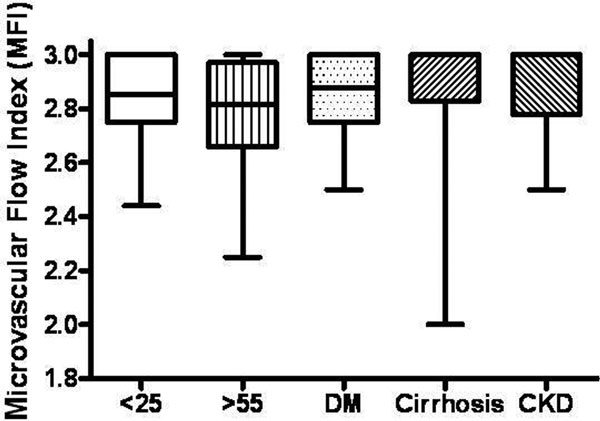


All DM patients were type 2, with mean glycated haemoglobin (HbA1c) of 8.8% (SD 1.7%). Seventeen cirrhotic patients were Child- Pugh-Turcotte score A and one was score B. For CKD, the mean estimated glomerular filtration rate was 11.5 ml/minute (SD 2.9). Median microvascular flow index (MFI) was 2.85 (IQR 2.75 to 3.0) for participants aged <25, 2.81 (2.66 to 2.97) for those aged >55, 2.88 (2.75 to 3.0) for those with DM, 3.0 (2.83 to 3.0) for those with cirrhosis and 3.0 (2.78 to 3.0) for those with CKD (*P *for difference = 0.14). There were no significant differences in the proportion of perfused vessels and perfused vessel density between the groups. See Figure 1.

## Conclusion

Older age, diabetes, and chronic kidney and liver disease need not be considered confounding factors for comparison of SDF microcirculatory parameters in the critically ill.
